# New Insights into the Co-Occurrences of Glycoside Hydrolase Genes among Prokaryotic Genomes through Network Analysis

**DOI:** 10.3390/microorganisms9020427

**Published:** 2021-02-19

**Authors:** Alei Geng, Meng Jin, Nana Li, Daochen Zhu, Rongrong Xie, Qianqian Wang, Huaxing Lin, Jianzhong Sun

**Affiliations:** Biofuels Institute, School of the Environment and Safety Engineering, Jiangsu University, Zhenjiang 212013, China; jinmeng26@hotmail.com (M.J.); Muzinaya@hotmail.com (N.L.); dczhucn@hotmail.com (D.Z.); rrxie@ujs.edu.cn (R.X.); wqq@ujs.edu.cn (Q.W.); linhuaxingUJS@outlook.com (H.L.)

**Keywords:** glycoside hydrolases, prokaryotes, co-occurrence, network, Gephi

## Abstract

Glycoside hydrolase (GH) represents a crucial category of enzymes for carbohydrate utilization in most organisms. A series of glycoside hydrolase families (GHFs) have been classified, with relevant information deposited in the CAZy database. Statistical analysis indicated that most GHFs (134 out of 154) were prone to exist in bacteria rather than archaea, in terms of both occurrence frequencies and average gene numbers. Co-occurrence analysis suggested the existence of strong or moderate-strong correlations among 63 GHFs. A combination of network analysis by Gephi and functional classification among these GHFs demonstrated the presence of 12 functional categories (from group A to L), with which the corresponding microbial collections were subsequently labeled, respectively. Interestingly, a progressive enrichment of particular GHFs was found among several types of microbes, and type-L as well as type-E microbes were deemed as functional intensified species which formed during the microbial evolution process toward efficient decomposition of lignocellulose as well as pectin, respectively. Overall, integrating network analysis and enzymatic functional classification, we were able to provide a new angle of view for GHs from known prokaryotic genomes, and thus this study is likely to guide the selection of GHs and microbes for efficient biomass utilization.

## 1. Introduction

Microbes play a critical role in element recycling on earth, due to their robust catabolic capacities. The prokaryote is a simple yet essential division of microbes, and it has various metabolism patterns, which could generally be classified as autotroph and heterotroph. Large organic molecules from biomass, such as carbohydrates and proteins, can be readily decomposed by particular heterotrophic prokaryotes to produce water, carbon dioxide, nitrogen, etc., which could be reused by other organisms [1].

It is carbohydrate-active enzymes (CAZymes) that endow living organisms, including prokaryotes, with the capability of carbohydrate synthesis and decomposition, the latter of which is actually one of the most prevalent processes of carbon assimilation and energy utilization in heterotrophs. According to the CAZy database (http://www.cazy.org, updated on 25 July 2020), CAZymes are composed of diverse families of glycoside hydrolases (GHs), glycosyltransferases (GTs), polysaccharide lyases (PLs), carbohydrate esterases (CEs), and auxiliary activities (AAs). These highly differentiated CAZymes participate in the reactions of breakdown, biosynthesis, or modification of carbohydrates, which are prevalent in organisms. The CAZy database has therefore become an essential reference for the annotation of newly discovered sequences of nucleic acids and proteins [2]. Among these CAZymes, GHs are the core enzyme categories for the degradation of structural polysaccharides, and also are important industrial enzyme categories for food manufacturing, biofuels production, textile polishing, paper making, and so on [3].

Based on protein sequences, GHs are currently divided into 168 families (including seven deleted families), which is nearly four times larger than the total number of glycoside hydrolase families (GHFs) that were proposed at first in 1991 [4]. Owing to the fast development of DNA sequencing technology in the past decade, more and more genomic/metagenomic sequences have been obtained [5]. Garron and Henrissat [6] recently reported the linear increment of total numbers of known GHFs over time. Furthermore, several large GHFs were subsequently divided into a series of subfamilies [7,8]. Due to the much smaller sizes of prokaryotic genomes than those of eukaryotes, a remarkably larger number of prokaryotic genomic sequences were achieved than those of eukaryotes (17,923 vs. 293 as noted in the CAZy database). Based on this relatively large number of samples, it is possible to perform statistical analysis of GH genes among prokaryotic genomes.

Most importantly, there is a prevalent co-evolution relationship, not only between functional relevant genes [9,10] but also between their transcription levels [11,12], which maintains the structure of ecological and molecular networks. This co-evolution relationship may also exist among GH genes, which collaboratively participate in polysaccharide decomposition. In fact, Berlemont and Martiny [13] have recently demonstrated the correlation between fluctuations of microbial community structure and GH potential for carbohydrate utilization among 13 ecosystems, using GH information from thousands of metagenomic datasets. The connection between GHs and ecological network is thus clear; nevertheless, the molecular networks for GHs remain largely elusive. It is fortunate that primary enzyme functions for an array of GHFs have previously been described according to the CAZy database [14]. Together with these functional profiles, correlation analysis between different GHFs may contribute to revealing the evolutionary relationships in the molecular network of GHFs, as well as their potential synergistic effects in polysaccharide decomposition. Berlemont and Martiny [15] also surveyed the known sequenced bacterial genomes and suggested the linkage between GHF contents and bacterial functions in polysaccharide deconstructions; however, archaea were excluded.

In this study, GH profiles from more than 17,000 prokaryotic genomes were retrieved from the CAZy database, and subsequently parsed and organized for statistical and correlation analysis. The difference in GHF distribution between bacteria and archaea was compared. Software Gephi [16] was employed to build the modular structure for the community of GHFs, where closely related GHFs were clustered and distinguished from the others. Meanwhile, enzymatic function profiles were also included so as to classify these GHFs into functional relevant groups. Finally, twelve functional relevant groups were identified, and a map for the evolution of GHFs was illustrated for the prokaryotes. Our work may help to better understand the evolutionary relationship among these GHFs and provide new strategy for the utilization of carbohydrate resources.

## 2. Methods

### 2.1. Information Acquisition and Matrix Construction

The GH family information for each prokaryotic genome was retrieved from the CAZy database (Data Sheet 1), and subsequently parsed using a Python script to form a matrix presenting the gene numbers of each GH family for every prokaryotic strain. This matrix was subsequently trimmed to leave a unique strain for each species, so as to eliminate the unwanted bias, as some species may be composed of many genome-sequenced strains, while, in contrast, others might only have unique ones. A derived matrix, named aveF, which is composed of the average gene numbers (rounding to the nearest integer) of each GHF for each species, was constructed (Table S1). By comparison, a derivative matrix, named minF (Table S2), was composed of the unique strains which harbor the minimum numbers of GHFs for each species, while derivative matrix maxF (Table S3) was composed of those which harbor the maximum. If there were two strains which harbored equal family numbers in the same species, the one which came first in the ordinal number was chosen for the minF matrix according to their alphabet strain names; conversely, the one which came last was chosen for the maxF matrix. The alphabet ordinal treatment rather than a treatment following the ordinal of total gene numbers produced a relatively random selection of strains from each species. Strains which had no defined species names were also trimmed to leave a unique strain for each genus and included in the three matrices, using the manners described above.

### 2.2. Calculations on the Distribution of GHs

The occurrence frequency for specific GH families was determined as the numbers of species that have at least one gene divided by the total number of species, as listed in matrix aveF (Table S1), which represented the average profiles of each species. Average gene numbers for specific GH families were defined as the total number of GH genes from specific GH families divided by the counts of species which possess at least one gene belonging to that family (rather than the total number of species, so as to eliminate the bias originating from the drastic variations in occurrence frequency, ranging from 0 to 91.7%). Both the occurrence frequency and average gene numbers were calculated separately for bacteria and archaea using data from matrix aveF.

### 2.3. Network Construction and Functional Group Classification

For microbial network construction, the matrix aveF (Table S1) was subjected to the Spearman’s rank correlation analysis between every two species, employing an online OmicShare^TM^ tool (www.omicshare.com/tools, on 28 August 2020). Similar GHF patterns were identified in cases where the correlation coefficient (ρ) was larger than 0.8 and statistically significant (*p* < 0.001). These strongly correlated species were used to build a weighted undirected microbial network with a modular resolution of 1 (finest), employing Gephi 0.9.2, by which a set of network topological properties (e.g., degree, modularity, clustering coefficient, and average path length) were also calculated.

For GHF network construction, we used a lower cut-off value of correlation coefficients, but a three-fold checked strategy for edge validation. The aforementioned three matrices were separately subjected to the Spearman’s rank correlation analysis between every two GHFs, employing the online OmicShare^TM^ tool. Co-occurrence of GHFs was validated if the correlation coefficient (ρ) was larger than 0.45 and statistically significant (*p* < 0.001), whichever matrix was employed. Interestingly, for these validated co-occurrence events (that is, for a given pair of GHFs), most of the correlation coefficients that were calculated based on the aveF matrix were very close to those of the minF and maxF matrix. Correlation coefficients larger than 0.4 suggested at least moderate-strong correlations. We raised the cutoff value to 0.45 so as to hold the confident positive correlations and shake off the ambiguous correlations, which might result from various combinations of strains harboring quite different numbers of genes for each GH family. Co-occurrence events were considered to be valid only if their correlation coefficients based on all of the three matrices were larger than 0.45.

Since most of the paired differences in these correlation coefficients were very small, the aveF-derived dataset was harnessed to construct the GHF network, employing Gephi 0.9.2 similarly to the microbial network described above, but only those solid relationships (always ρ > 0.45 in the calculations based on the three matrices) were involved. The nodes in the constructed network represent the GHFs, whereas the edges (viz. connections) correspond to an at least moderate-strong and significant correlation between nodes. Functional categories were firstly classified based on the modules detected by Gephi, which generated nine categories, and each of them was labeled with a unique color. In order to better interpret the network, three out of the nine categories were further subdivided into six categories according to the primary enzyme functions of the GHFs, which were reported in literature and subsequently collected in the CAZy database (Table S4). Briefly, GHFs that are known to be mutually involved in hydrolysis of specific natural substrate were clustered into the same category (Table 1 and Table S4). Pectin is a minor component of lignocellulose, but is an extremely complex polysaccharide, which may involve tens of kinds of CAZymes [17]. Hence, for the biggest module, pectin-active GHFs were highlighted and subclassified in group B, leaving the left GHFs within the module group A, which were active on bulk lignocellulose components. Similarly, in one module, hexosamine active GHFs (group G) were separated from glycosidases (group H), and in another module, GHFs (group K) that may be active on fungal cell wall polysaccharides (including alpha-1,3-glucan, beta-1,3-glucan, chitosan, and polygalactosamine) were distinguished from those active on bulk lignocellulose components (group L). GH144 is unique in hydrolysis of cleavage of β-1,2-glucan, which is related to bacterial infection or symbiosis [18], and thus was not grouped. Microbes that possess GHs from at least two GHFs from group X are thus termed as type-X microbes (Table 1 and Table S5), where X represents specific functional categories. 

### 2.4. Heatmap Construction

The numbers of GH genes within each aforementioned functional group were summed separately for each species in the aveF matrix to form a new matrix, named aveG (Table S5). The latter was then handled firstly by taking the square root of each number, and the row data were then further normalized into a Z-score. These transformed data were used to construct a heatmap, which reflects the distributions of total gene numbers for each functional group throughout all of the species, also employing the online OmicShare^TM^ tool. Specifically, a subset of aveG was highlighted for another heatmap, which involves exclusively genome-sequenced cellulolytic bacteria as reported in the literature, with updates [19]. 

### 2.5. Co-Evolution Analysis

Strongly correlated pairs of GHFs were subjected to co-evolution analysis using MirrorTree (http://csbg.cnb.csic.es/mtserver, on 7 December 2020). Briefly, two randomly selected protein sequences that belong to a pair of specific GHFs were submitted to the online server of MirrorTree using the default parameters, and homologs were automatically assigned for mirror tree construction [20]. Correlation coefficient among the inter-protein distances in both families was used to discover co-evolution events.

## 3. Results

### 3.1. GH Information Acquisition and Matrix Construction

GH information in prokaryotic genomes was acquired from the CAZy database, and subsequently trimmed for bias elimination as well as statistical analysis. A total number of 17,375 items of GHF-distribution information for known prokaryotic genomes were retrieved from the CAZy database. Matrix aveF that represents the average gene numbers of each GHF for each species was constructed (Table S1). Meanwhile, matrix minF that represents the core and essential GH profiles for each prokaryotic species (Table S2), and matrix maxF that represents the possible largest stretches in GHFs for prokaryotes (Table S3), were also constructed for comparison. These three matrices were composed of 258 rows of archaea and 3986 rows of bacteria, distributed in 154 (151 for minF) columns (GHFs). Nearly twenty GHFs were not included in these matrices, either because the families have been removed from the CAZy database or because of the absence of these GHFs in prokaryotes.

### 3.2. Occurrences of Genes from Various GHFs 

**Figure 1:**
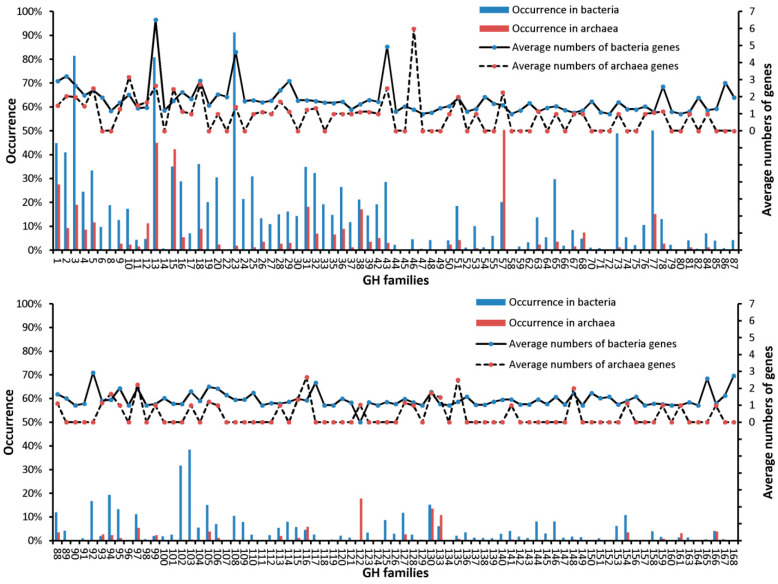
Occurrence frequencies and average numbers of genes for various glycoside hydrolase families (GHFs) in prokaryotes. The calculations were based on the aveF matrix, and rare GHFs for the prokaryotes are not shown. Average number of GH genes was calculated according to the total number of genes in a specific GHF divided by the count of species which possess at least one gene for this GHF.

Bacteria dominated over archaea in most GHFs (134 out of 154) in terms of both occurrence frequencies and average gene numbers, as shown in Figure 1, which was illustrated based on the average GH profiles of the prokaryotes (matrix aveF). Specifically, the occurrence of GH genes from 144 families was higher in bacteria than in archaea, and in 64 families the former was at least 5% higher than the latter. Furthermore, 85 families were exclusively found in bacteria. In addition, the average gene numbers in 95 families were always one copy larger in bacteria than those in archaea. The top five GHFs for bacteria were GH3, GH13, GH23, GH73, and GH77, with the maximum occurrence frequency as high as 89.3% in GH23. 

Nevertheless, several GHFs were indeed prone to show up in archaea. The frequency of GH genes from 10 families was higher in archaea rather than bacteria, and in four families the former was always at least 5% higher than the latter (GH12, GH15, GH57, and GH122), with GH122 exclusively found in archaea. The top five GHFs for archaea were GH1, GH13, GH15, GH31, and GH57. Moreover, the average gene numbers in five families were always one copy larger in archaea than those in bacteria (GH10, GH46, GH116, GH122, and GH135). Generally, GHs showed up in 97.9% of the prokaryotic genomes that were collected in the CAZy database, and GH1, GH13, GH15, and GH57 seem to be common between bacteria and archaea, with the occurrence frequency no less than 18%.

### 3.3. Co-Occurrence and Network Analysis of GHs

Different extent of co-occurrences was found among the 154 GHFs. Calculation based on the matrix aveF suggested the existence of strong correlation (Spearman’s ρ > 0.6, *p* = 0) among 24 family pairs, which accounted for 0.2% in all of the possible family pairs, meanwhile moderate-strong correlation (ρ between 0.4 and 0.6, *p* < 0.001) was found among 317 family pairs, which accounted for 2.7%. Correlation analysis was also performed based on matrix minF and maxF, which represent the two extremes of each species in terms of total number of GHFs. Interestingly, for most of the validated co-occurrence events (ρ > 0.45, *p* < 0.001), correlation coefficients that were calculated based on the aveF matrix were very close to both of those of the minF and maxF matrix. These paired differences had a mean of 0.01, with a standard deviation of 0.01. However, a few higher variations were observed in the range of 0.05 to 0.08. We therefore raised the cutoff value of correlation coefficients from 0.4 to 0.45 so as to hold the confident positive correlations and decrease the ambiguous ones, the latter of which might occur during the random selection of a strain from particular species for matrix construction. Since the aveF matrix represents the average GH profiles for the prokaryotes, and the correlation coefficient between each family pair was quite similar to the one based on minF, as well as that of maxF, the former dataset was employed for the subsequent network construction. 

**Figure 2:**
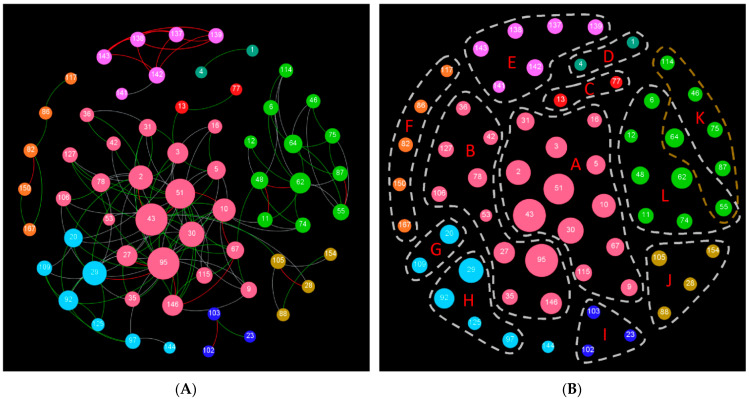
(**A**) Co-occurrence network for the GHFs as constructed by Gephi and (**B**) the classified functional categories. Solid circles (nodes) represent various GHFs, which are divided into nine modules, distinguished with colors. Red lines (edges) suggest strong correlations (Spearman’s ρ > 0.6), and green as well as grey lines infer moderate-strong correlations (0.5 < ρ < 0.6 as well as 0.45 < ρ < 0.5, respectively). An array of functional categories are clustered and marked with dash lines.

A correlation network was successfully built by employing Gephi with the aveF dataset. As shown in Figure 2A, the network shows a good modular structure with a modularity index of 0.532 (values larger than 0.4 suggest that the network has a modular structure). The average network distance between all pairs of nodes was 3.0, with a diameter of eight edges, and the average clustering coefficient was 0.66. The network is composed of 63 nodes (GHFs) and 160 edges (connections), with nine differentiated modules. The largest module possesses as much as 22 nodes, whereas the two smallest modules each have only two nodes. Interestingly, a module with several strong connections was observed among GH137, GH138, GH139, GH142, and GH143.

### 3.4. Network Analysis of Microbes

**Figure 3:**
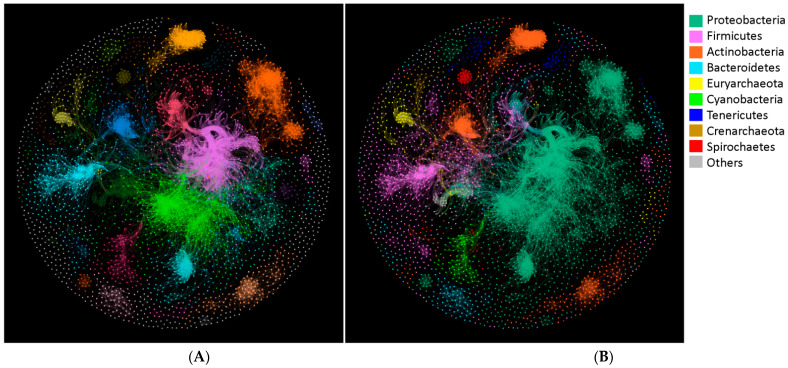
Network analysis of microbes using Gephi. (**A**) Primary modules were rendered with different colors and (**B**) microbes were distinguished by colors according to their affiliated phyla.

Different extent of correlation was found among the 4244 species listed in matrix aveF (Table S1), and very strong correlations (Spearman’s ρ > 0.8, *p* < 0.001) were detected between 27,926 microbial pairs, which accounted for 0.3% out of all possible pairs of microbes. The microbial network was thus constructed based on these very strong correlations, involving 3268 nodes (77% of the species listed in Table S1) and 27,926 edges. The average network distance between all pairs of nodes was 5.9, with a diameter of 19 edges. The average clustering coefficient was 0.59 and the modular index was 0.815, indicating that the network has a good modular structure (Figure 3A). Most modules were each specifically adjunct to a primary microbial phylum (Figure 3B); however, most phyla tend to consist of microbes from at least two modules, indicating that the GHF patterns could be divergent for specific phylum, but be conversed within a subset of the phylum. For example, the biggest phylum, Proteobacteria, was composed of microbes from 50 modules; however, Proteobacteria was predominant (>95%) in each of the top six out of the 50 modules, as well as in most of the left small modules. Similarly, Bacteroidetes was composed of microbes from 25 modules; nevertheless, up to nineteen modules were exclusively composed of Bacteroidetes, including the top three out of the 25 modules, suggesting the close evolutionary relationships among microbes within these modules, in terms of GHF profiles. By contrast, a few heterogeneous modules did exist. For instance, two modules were composed of 37 to 45% Firmicutes, 16 to 29% Proteobacteria, and 26 to 47% other phyla.

### 3.5. Classification of Functional Categories

Twelve functional categories (group A to L) were identified from the network (Figure 2B and Table 1), after a round of modular identification, as well as a subsequent round of functional classification based on the primary enzyme functions of the GHFs (Table S4) as described in Methods. Several groups were closely related, not only in function but also in their microbial distributions. Both group A and L consist of GHs active in the decomposition of bulk lignocellulose components, such as cellulose, glucuronoarabinoxylan, xyloglucan, and β-glucan. In addition, the microbial collection with GHs from group A (type-A microbial collection) covered more species than those of type-L (2281 vs. 338 species), and the type-L collection is actually a subset of that of type-A (Table 1 and Figure S1A). Group B, E, and J involve GHs which are active in degradation of pectic polysaccharide, but with some differences: group B and E are specifically active in debranching of rhamnogalacturonan II, while group J is more active in hydrolysis of the main chain of pectic polysaccharide, including homogalacturonan, rhamnogalacturonan I, and rhamnogalacturonan II. Moreover, the type-B cluster covered most species of the type-J cluster (86%), and the latter covered most of the type-E cluster (85%). The type-E collection is actually a subset of the type-B collection (Figure S1B).

Other groups were more loosely connected than the aforementioned groups. Group D and H contain various glycosidases, and their corresponding microbial collections were partially overlapped (less than 30%, Figure S1C). GHs from group C, F, G, I, and K were each active toward starch, cell wall polysaccharide from red algae, oligohexosamine, peptidoglycan, and fungal cell wall, respectively. In addition, GH55 and GH64 (primarily endo/exo-β-1,3-glucanases) were shared by group K and L because of their functional associations, while GH144 was not assigned in any categories because of its unique functions (cleavage of β-1,2-glucan, which is related to bacterial infection or symbiosis [18]). Although GH27 and GH36 might not be easy to distinguish [21], they had little influence on the functional classification, as they belong to the same functional category (group B).

The phylum distribution varied throughout the 12 functional categories (Table 1). Both type-A and -B microbes dispersed predominantly in four phyla, viz. Actinobacteria, Bacteroidetes, Firmicutes, and Proteobacteria, with frequencies varying from 23% to 88%. Similarly, type-C, -D, -G, -H -J, and -L microbes were distributed mainly in at least two of the four phyla. By comparison, type-F microbes assembled in phyla Bacteroidetes and Planctomycetes. Type-I microbes were found mostly in phyla Cyanobacteria and Proteobacteria. Type-E microbes were mainly composed of Acidobacteria and Bacteroidetes. Type-K microbes were mainly composed of Acidobacteria and Actinobacteria. The frequencies of type-D, -E, -F, and -L microbes (≤38%) detected in specific phylum were apparently much lower than those of other types of microbes (up to 100%). In addition, type-A, -B, -C, -D, -H, -I, -J, and -L existed in bacteria as well as archaea and, by contrast, other groups could be found exclusively in bacteria.

### 3.6. Heatmap Illustration of Gene Doses in Various Functional Categories

**Figure 4:**
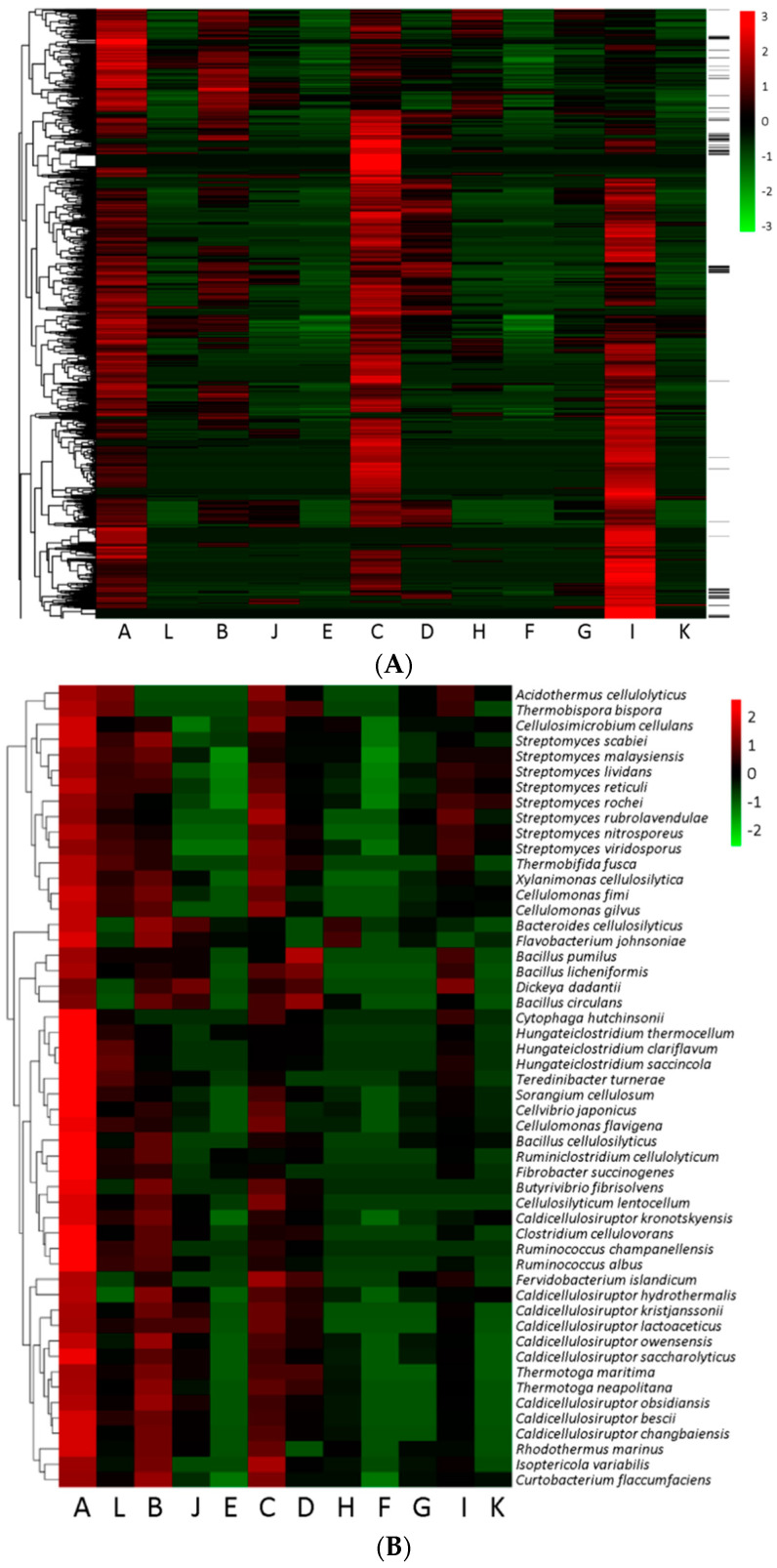
(**A**) Heatmap analysis of the gene doses for the 12 functional categories throughout the prokaryotes and (**B**) the known cellulolytic species. The black lines on the right (**A**) indicate archaea species. The total number of genes within specific functional categories was firstly transformed by taking their square roots, and then normalized using the Z-score method. The calculations were based on the matrix aveF.

Figure 4A depicts the relative abundance of GH genes for the prokaryotes throughout the 12 functional categories. Despite being in minority, archaea contributed to the co-occurrence of GHFs (Figure 4A and Table 1). Generally, in most species the A, C, and I groups consisted of more GH genes than other groups, and were more prevalent throughout the prokaryotic community, even though the latter two groups involved only two or three GHFs (Table 1). As compared among function-related groups, prokaryotes tend to share more GH genes from group A rather than L for the uptake of lignocellulose-derived carbohydrates. Similarly, most prokaryotes also succeeded in collecting more GH genes from group B rather than J and E for the assimilation of pectin-derived carbohydrates. The GH distribution exhibited somewhat complementary phenomena between group D and H, inferring the alternative sources of these GHs.

Heatmap was also employed to illustrate the GH distribution among the known genomes of cellulolytic bacteria. As compared to Figure 4A, Figure 4B clearly showed the enrichment of lignocellulolytic and pectinolytic groups (A, L, B, J, and E). Generally, the abundance of genes in group A was still higher than that of group L, and the abundance of genes in group B, J, and E decreased in their order in most species. In addition, the two prevalent groups (C and I) became less pronounced in Figure 4B than those of Figure 4A.

### 3.7. Co-Evolution Analysis

Quite different extent of co-evolution relationships was observed among these GHFs. Firstly, several significant evidences (*p* ≤ 0.000001) of very strong co-evolution relationships were observed among the GHFs within group E. According to the results of MirrorTree analysis, very strong correlations (*r* > 0.9, 11 < *n* < 17) were found among GH137, GH138, GH139, GH142, and GH143, except for the pair of GH 139 and GH142 (*r* = 0.138), suggesting the existence of strong co-evolution relationships among most of these GHFs, which primarily exist in Bacteroidetes. The relatively low n values were mainly due to the low occurrences of these GHFs (Figure 1). Figure S2 is an example of a mirror tree between GH137 and GH138, and other mirror trees among these above mentioned GHFs were quite similar to it. Secondly, evidences of strong co-evolution were also detected in other groups but in a dispersed manner. For instance, strong correlation was found between GH102 and GH103 (*r* = 0.932, *n* = 317) in Proteobacteria. This phenomenon was also observed between GH28 and GH105 (*r* = 0.744, *n* = 71), between GH10 and GH67 (*r* = 0.688, *n* = 98), and between GH43 and GH51 (*r* = 0.602, *n* = 101) in several phyla of bacteria. Thirdly, moderate-strong or weak correlations were also identified in several pairs of GHFs, for example, GH1 and GH4 (*r* = 0.480, *n* = 467), as well as GH48 and GH62 (*r* = 0.078, *n* = 5).

## 4. Discussion

GHs are essential for nearly all of the prokaryotes (97.9%), whereas their distributions are quite uneven. The co-occurrence of GHs from various families not only lies in their coincidence, but also in their inevitability. The correlational network analysis enables the global view of the relations among more than a hundred of GHFs, which may reveal new insights into the interactions and evolutionary relationships among GHs.

Most GH genes were prone to occur in bacteria rather than archaea, in terms of occurrence frequencies as well as average gene numbers (Figure 1). It is known that some archaea live in extreme environments (high temperature, high pressure, or high salt), and are well adapted to nutrient-limiting ecological niches, while other archaea have evolved in microbial communities to be specialized in the assimilation of the metabolic products of bacteria, i.e., formate, acetate, CO_2_, and H_2_ [22]. That is why GH genes were more frequently found in bacteria. Indeed, some archaea were reported to grow on complex carbohydrates, but these were in the minority [23,24]. Genes of GH122 were exclusively found in archaea, particularly of phyla Crenarchaeota and Euryarchaeota, demonstrating that this GH probably originated from archaea, and might not be well adapted to bacteria, thus not widely spread in the microbial community, and vice versa for the 85 bacteria-specific GHFs. That is because gene adaptation to the host is apparently a key process for gene gains through lateral gene transfer (LGT) [25,26]. This specificity is also observed in a well characterized GHF, GH7, which exclusively exists in eukaryotes, suggesting an ancestral specialization event [15]. In addition, despite being in minority, archaea did contribute to the co-occurrence of GHFs (Figure 3A and Table 1).

Co-occurrences might result from compromises between synergization and replacement in enzyme functions (probably in the form of gene gain and loss), as well as the differences in gene adaptation to host. Quite different extent of co-occurrences of GHs from various families was observed in prokaryotes. On one hand, synergization or replacement in enzyme functions influences the co-occurrences. For example, strong co-occurrences were found in most combinations among GH137, GH138, GH139, GH142, and GH143 (ρ > 0.6, Figure 2A), for the possible reason that they synergize in debranching of Rhamnogalacturonan II [27]. By comparison, weak or moderate-strong co-occurrences were observed among GH10, GH11, and GH30 (ρ < 0.47), probably attributing to their similar and alternative functions (primarily endoxylanases, although acting on different types of sites of glucuronoarabinoxylan [28]). On the other hand, co-occurrences may also be affected by gene adaptation to the host during LGT. Prokaryotes possess the ability to acquire new genes through LGT, but will lose the useless ones [26]. If a newly transferred GH is well adapted to some organisms but not to others, this would lead to a drop in co-occurrences for its GHF with other relevant GHFs, the latter of which may have quite different adaptation patterns. It has been proposed that co-evolution exists among functional relevant genes so as to maintain the structure of ecological and molecular networks [9,10]. Moreover, Kim and Price [29] show that genes preferentially co-occur when they either encode physically interacting proteins or are co-expressed. Our data show the existence of co-occurrences as well as co-evolution among various GHFs, and well support these perspectives. Furthermore, some GH genes have been found to cluster together in tight linkage, which is obviously the result of co-evolution. For example, twelve GH genes that are involved in biomass decomposition cluster together and some of them are co-expressed in *Clostridium cellulolyticum* [30]. 

It should be noted that the occurrence frequencies of GHFs documented in this work were unweighted values, which were different from naturally occurring frequencies in specific microbial communities, and so were the co-occurrences among them. However, these occurrences and co-occurrences might to some extent reflect the spread potential of GHFs among prokaryotes. As expected, the sixty-three nodes in Figure 2 covered at least the top 10 GHFs among the metagenomes from a series of niches, such as rumen [31], termite gut [32], freshwater and soil [33], etc., and therefore might represent the hotspot GHFs among various microbial communities. Moreover, Berlemont and Martiny [15] recently surveyed the known sequenced bacterial genomes and found the prevalence of GH1, GH2, GH3, and GH4 in bacteria for oligosaccharides decomposition, as well as the richness of GHFs in Bacteroidetes for the hydrolysis of a series of components of plant cell wall polysaccharides. Furthermore, the polysaccharides degraders were found to possess more oligosaccharides-degrading genes than those of the non-degraders. Our results match well with these findings, and the network analysis further enabled us to identify 12 functional categories of GHFs, as well as two groups of functional intensified microbes for polysaccharides decomposition.

Network visualization is a powerful method for the understanding of complex relationships throughout a community or group [34]. Recently, software Gephi and iGraph have been successfully applied in resolving and illustrating complex microbial communities [35,36], where close relevant members constitute a module. These studies used a higher cutoff value of Spearman’s coefficients (ρ = 0.5 or 0.6, respectively) in order to simplify the huge microbial network, while we chose a lower yet still valid cutoff value of 0.45 (*p* < 0.001) so as to isolate more useful information among the 168 GHFs. Network analysis also enables member classification in a community. Barberan et al. [35] successfully distinguished the habitat specialists from the generalists among the soil bacterial communities by using network analysis. Yang and Wang [37] recently identified the positively and negatively responding genes as well as microbial phyla in eutrophic lake water when exposed to a cationic surfactant by employing network analysis. Similarly, twelve functional categories were classified in our work according to an integration of network visualization (module identification) and the primary functions of various GHFs (Figure 2B, Table 1 and Table S4). These functional categories connect not only the co-occurring GHFs, but also their hosts (species), and therefore present comprehensive profiles for GHs as well as functional prokaryotes.

GHs in the categories of lignocellulose decomposition are obviously enriched in cellulolytic bacteria; nevertheless, the presence of these GHs does not necessarily suggest the cellulolytic capability. As in the heatmap illustration of GH distributions within the 12 functional categories, the chances of GHs present in the categories of lignocellulose decomposition (group A and L), as well as pectin decomposition (group B, J, and E), were remarkably higher in cellulolytic microbes (Figure 4A,B), inferring the potential roles of these GHs in lignocellulose decomposition. Figure 4A depicts the presence of two extremely abundant functional groups (A and C), which is consistent with the fact that lignocellulose and starch were among the most plentiful carbohydrate substrates for prokaryotes [38]. Another rich category, group I, is involved in peptidoglycan cleavage for cell division, which is an essential and prevalent process of cell growth [39]. However, the abundant and prevalent characteristic of group A does not necessarily suggest the cellulolytic capability of the corresponding species (Figure 4A). Because the production of extracellular β-1,4-endoglucanase as well as cellobiohydrolase is crucial for cellulose degradation [40], without these two enzymes microbes harboring genes of group A and L could just be possibly located in the downstream of lignocellulose utilization in a microbial community (viz., assimilation of various oligosaccharides which are released by biomass degraders). If this is true, most prokaryotes can live on lignocellulose along with biomass degraders. As a matter of fact, ample non-cellulolytic bacteria have been found to grow well on cellulosic substrates by co-culturing along with cellulolytic bacteria [41,42], as did those non-cellulolytic ones in cellulolytic microbial communities [43].

**Figure 5:**
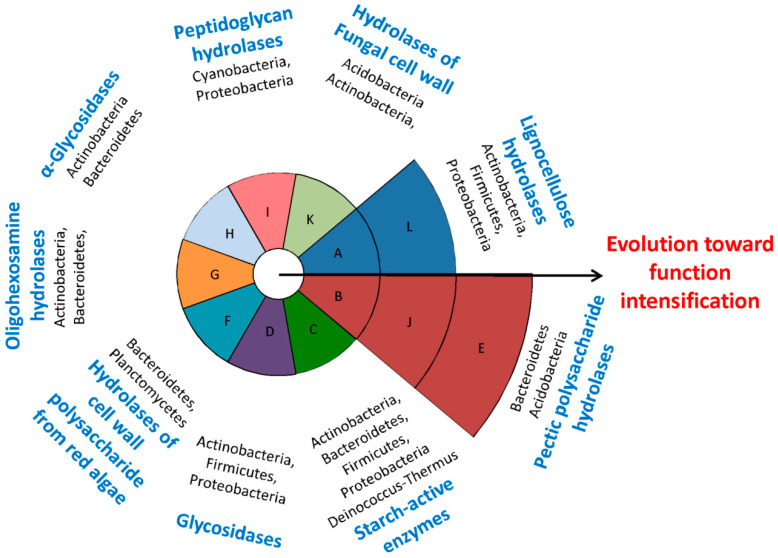
Overview of the functional categories and their corresponding dominant microbial phyla, as well as their relationships of progressive enrichment of specific GHFs.

From an evolutionary point of view, a progressive enrichment of GHFs is found among several types of microbes. As demonstrated in Figure 5 and Figure S1, high proportions of overlaps were observed between Type-A and -L microbial collections, as well as among Type-B, -J, and -E microbial collections (80% to 100%). In other words, nearly all of the type-L microbes will harbor GHs of group A, and similarly most type-E microbes will harbor GHs of group B as well as J. Type-L (some Actinobacteria, Firmicutes, and Proteobacteria) and type-E microbes (primarily Bacteroidetes) thus represent functional intensified prokaryotes, which have progressively evolved to become outstanding species for the decomposition of lignocellulose and pectin, respectively. In fact, type-L and type-E microbes have continuously been proved to play leading roles in biomass degradation, such as *Hungeteiclostridium thermocellum* [44] as well as *Caldicellulosiruptor bescii* [45] for lignocellulose decomposition, and *Bacteroides thetaiotaomicron* [27] as well as *Flavobacterium johnsoniae* [46] for pectin degradation. By contrast, although sharing very relevant functions, the overlaps between type-D and -H microbial collections remained at low level (less than 44%). GHs belonging to these two functional categories might exist as alternatives in the process of microbial evolution. The reason behind the progressive enrichment of particular GHFs is probably that for group A and L, as well as group B, J, and E, each category is not sufficient for the full decomposition of the complex and recalcitrant substrates (lignocellulose [47] and pectin [17], respectively). In fact, pectin is recognized as the most structurally complex polysaccharides in nature, which involves tens of monosaccharides as well as linkages [17]. In contrast, for group D and H, GHs of each category may be already enough for the degradation of the less recalcitrant substrates (oligosaccharides). 

We herein propose a new strategy for efficient biomass utilization. Since both lignocellulose and pectin are components of plant cell walls, it is possible that co-culture of the functional intensified type-L and -E microbes may promote biomass decomposition, in regard to simultaneously hydrolyzing very complex polysaccharidic components. Although co-cultures of cellulolytic and non-cellulolytic bacteria have been studied extensively [41,42,48], a co-culture of type-E and type-L microbes toward efficient biomass utilization is never seen. Moreover, plenty of metagenomic studies have indicated the constant presence of type-E microbes (primarily Bacteroidetes) along with type-L microbes in cellulolytic microbial communities [43,49], but the contribution of these type-E microbes to biomass decomposition may be underestimated. To this end, a co-culture of type-L and -E microbes may help to address this question.

It should be noted that the microbial full decomposition of complex carbohydrates requires a series of CAZymes, such as GHs, CEs, PLs, and AAs, as well as some non-enzyme components, viz. carbohydrate-binding modules. Our work only investigated the network profiles of one of them, though the most diverse and essential one: GHs. Our results provide a new angle of view for GHs in prokaryotes and might guide the selection of GHs and microbes for efficient biomass utilization. Further work is needed to recruit more CAZyme categories for thorough analysis of the interactions and evolutionary relationships among different CAZyme categories.

## Figures and Tables

**Table 1:** GHF distributions and functional categories.

Groups	Ghfs	Profiles of the Enzymatic Functions	No. of Species (Phyla) Largely Fitted ^a^	No. of Species (Phyla) Partially Fitted ^b^	Sources ^c^	Predominant Phyla (No. of Species, Frequency) ^d^
A	3, 5, 9, 10, 16, 30, 31, 43, 51, 67, 115	Widely distributed GHs for the decomposition of bulk lignocellulose components, such as cellulose, β-glucan, and glucuronoarabinoxylan, xyloglucan	283 (12)	2281 (28)	m	Actinobacteria (516, 78%), Bacteroidetes (262, 88%), Firmicutes (337, 48%), Proteobacteria (895, 50%)
B	2, 27, 35, 36, 42, 53, 78, 95, 106, 127, 146	Widely distributed GHs for the debranching of pectic polysaccharides, specifically Rhamnogalacturonan II	154 (10)	1475 (24)	m	Actinobacteria (341, 53%), Bacteroidetes (202, 72%), Firmicutes (335, 48%), Proteobacteria (413, 23%)
C	13, 77	GHs for the decomposition or modification of starch	2007 (30) ^e^	/	m	Actinobacteria (457, 69%), Cyanobacteria (89, 98%), Deinococcus-Thermus (27, 100%), Proteobacteria (896, 50%)
D	1, 4	Glycosidases	844 (16) ^e^	/	m	Actinobacteria (222, 34%), Firmicutes (269, 38%), Proteobacteria (262, 15%)
E	137, 138, 139, 141, 142, 143	GHs for the debranching of pectic polysaccharides, specifically Rhamnogalacturonan II	37(1)	73 (4)	s	Acidobacteria (5, 38%), Bacteroidetes (52, 18%)
F	82, 86, 117, 150, 167	GHs for the decomposition of cell wall polysaccharides from red algae and seaweeds	13 (4)	43 (6)	s	Bacteroidetes (19, 6%), Planctomycetes (6, 17%)
G	20, 109	GHs for the decomposition of hexosamine	294 (10) ^e^	/	s	Actinobacteria (70, 10%), Bacteroidetes (162, 55%)
H	29, 92, 97, 125	α-Glycosidases	314 (9)	579 (14)	m	Actinobacteria (132, 20%), Bacteroidetes (221, 75%)
I	23, 102, 103	GHs for the decomposition of peptidoglycan	1080 (4) ^e^	1701 (9)	m	Cyanobacteria (74, 81%), Proteobacteria (1608, 90%)
J	28, 88, 105, 154	GHs for the decomposition of the main chain of pectic polysaccharides, including Homogalacturonan, Rhamnogalacturonan I, and Rhamnogalacturonan II	312 (11)	623 (15)	m	Acidobacteria (10, 77%), Bacteroidetes (144, 49%), Firmicutes (114, 16%), Proteobacteria (231, 13%)
K	46, 55, 64, 75, 87, 114	GHs for the decomposition of fungal cell wall polysaccharides, including alpha-1,3- glucan, beta-1,3-glucan, chitosan, and polygalactosamine	78 (2)	231 (9)	s	Acidobacteria (6, 46%), Actinobacteria (128, 26%)
L	6, 11, 12, 48, 55, 62, 64, 74	Supplemental GHs of group A for more efficient decomposition of bulk lignocellulose components, such as cellulose, beta-glucan, and glucuronoarabinoxylan, xyloglucan	93 (2)	338 (12)	m	Actinobacteria (201, 30%), Firmicutes (49, 7%), Proteobacteria (59, 3%)

^a^ No less than 75% GH families within each group was present. ^b^ At least two GH families within each group were present. ^c^ s, specific for bacteria and m, mutual for bacteria and archaea. ^d^ Based on the numbers of species which partially fitted their group patterns. The numbers of species, as well as the frequencies of occurrence, were indicated in the parentheses. ^e^ Perfectly matched their group patterns.

## Data Availability

Publicly available datasets were analyzed in this study. These data can be found here: http://www.cazy.org/Genomes.html (accessed on 18 February 2021).

## Supplementary Materials

The following are available online at https://www.mdpi.com/2076-2607/9/2/427/s1, Data Sheet 1: List of the website addresses for the GH information of prokaryotic strains. Figure S1: Venn diagrams showing the species distributions among different types of microbes, (A) group A and L; (B) group B, J, and E; (C) group D and H. Figure S2: Mirror tree illustration of the co-evolution between GH137 and GH138. Table S1: Matrix aveF. Table S2: Matrix minF. Table S3: Matrix maxF. Table S4: Primary functions for various GHFs in prokaryotes. L1 to L3 represent the three most frequently found enzyme functions in characterized enzymes, respectively. Table S5: Distributions of numbers of GH genes and families in terms of the 12 functional categories.
