# Supplementary figures and images for: New Insights into the Co-Occurrences of Glycoside Hydrolase Genes among Prokaryotic Genomes through Network Analysis
Source: Microorganisms. 2021 Feb 19;9(2):427. doi: 10.3390/microorganisms9020427 (PMC7922503; doi:10.3390/microorganisms9020427)

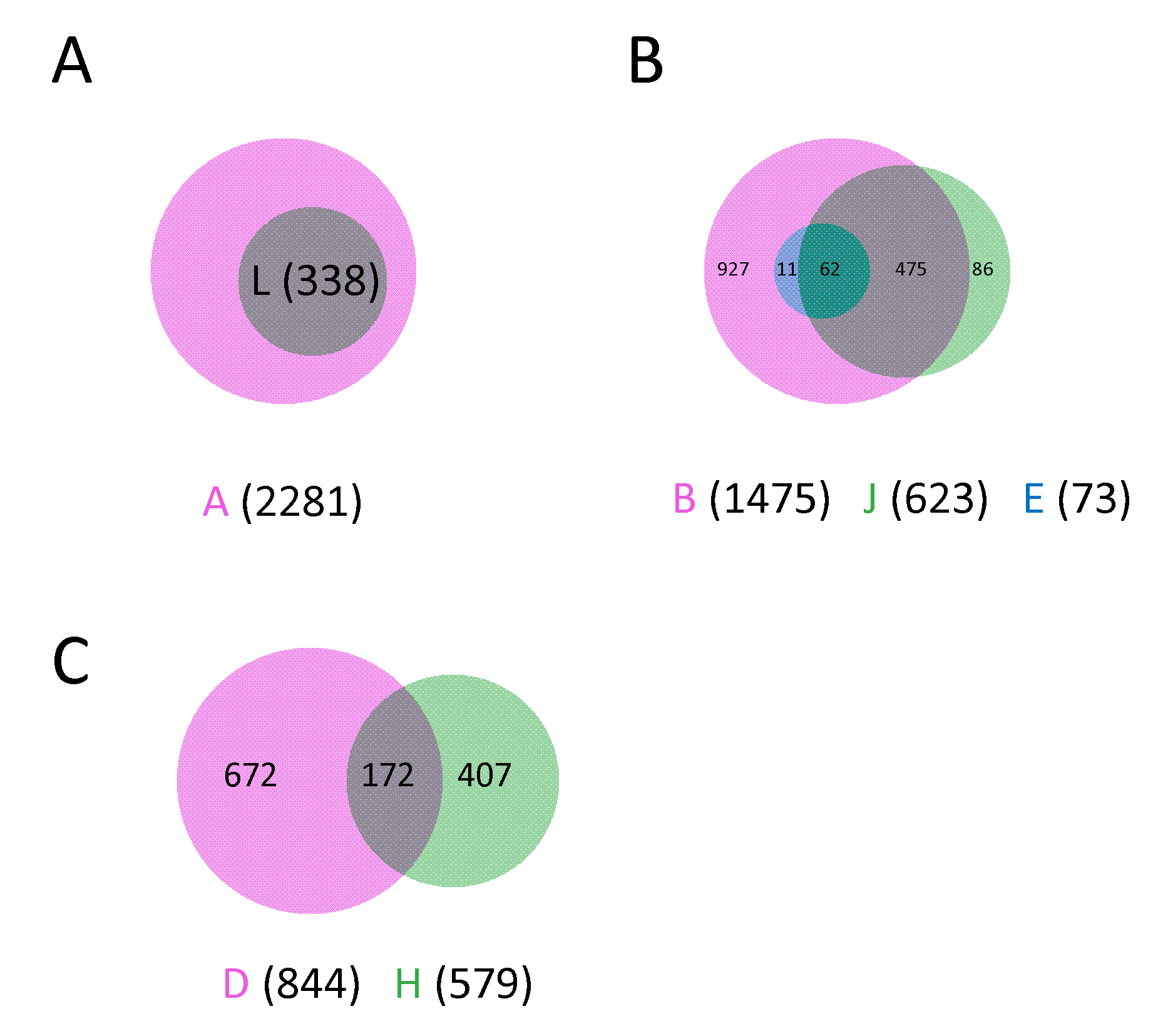

Supplement: Supplementary file 1 [file microorganisms-09-00427-s001.zip › suppl materials/Fig S1.tif]

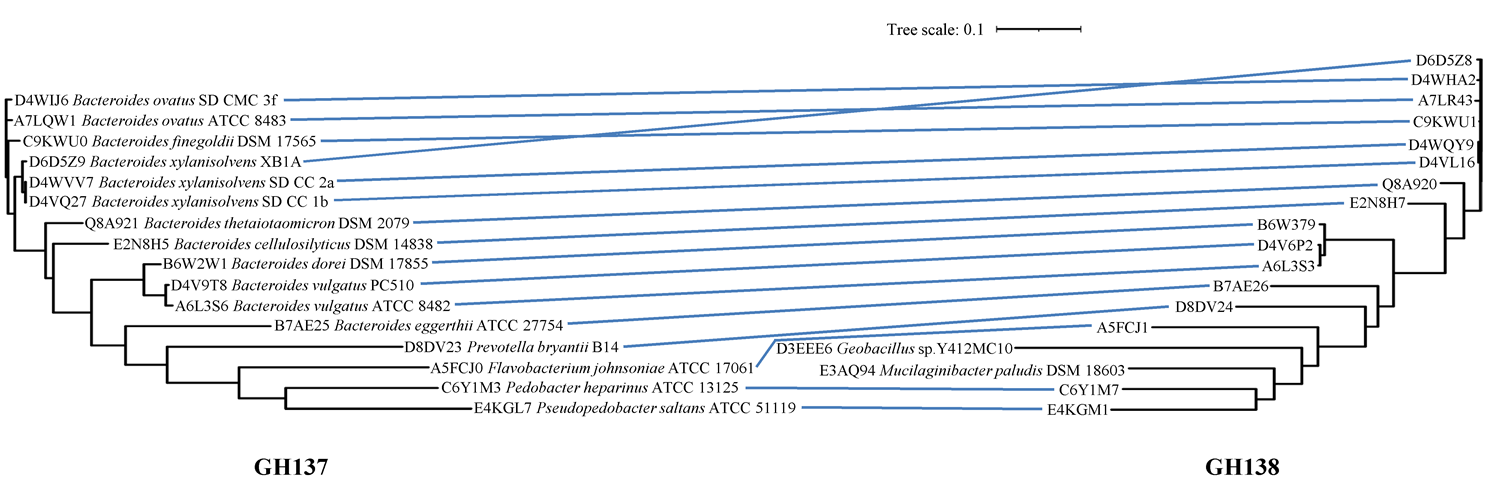

Supplement: Supplementary file 1 [file microorganisms-09-00427-s001.zip › suppl materials/Fig S2.tif]
